# Hypertension in Non-Type 2 Diabetes in Isfahan, Iran: Incidence and Risk Factors

**DOI:** 10.1155/2017/3132729

**Published:** 2017-12-20

**Authors:** Mohsen Janghorbani, Ashraf Aminorroaya, Masoud Amini

**Affiliations:** Isfahan Endocrine and Metabolism Research Center, Isfahan University of Medical Sciences, Isfahan, Iran

## Abstract

**Objective:**

To estimate the incidence of and risk factors for the development of hypertension (HTN) in people with T1D using routinely collected data.

**Method:**

The mean 16-year incidence of HTN was measured among 1,167 (557 men and 610 women) nonhypertensive patients with T1D from Isfahan Endocrine and Metabolism Research Center outpatient clinics, Iran. HTN was defined as a systolic blood pressure (BP) of 140 mm Hg or higher and/or a diastolic BP 90 mm Hg or higher and/or use of antihypertensive medications. The mean (standard deviation [SD]) age of participants was 20.6 years (10.5 years) with a mean (SD) duration of diabetes of 3.6 years (4.8 years) at registration.

**Results:**

The prevalence of HTN at baseline was 9.7% (95% CI: 8.2, 11.5). Among the 1,167 patients free of HTN at registration who attended the clinic at least twice in the period 1992–2016, the incidence of HTN was 9.6 (8.0 women and 11.3 men) per 1000 person-years based on 18,870 person-years of follow-up. Multivariate analyses showed that male gender, older age, higher triglyceride, and higher systolic BP were significantly and independently associated with the development of HTN in this population.

**Conclusion:**

These findings will help the identification of those patients with T1D at particular risk of HTN and strongly support the case for vigorous control of BP in patients with T1D.

## 1. Introduction

Hypertension (HTN) is the most important risk factor for cardiovascular disease and associated with increased risk of nephropathy [1, 2] and retinopathy [3–5] and is estimated to affect about one-third of patients with T1D [6] and is one of the well-established modifiable risk factors for cardiovascular disease mortality and morbidity [7–9]. Although some studies have examined the prevalence of HTN in patients with T1D in cross-sectional reports, mostly in developed countries, only a few studies have reported the results of a longitudinal analysis on the incidence of HTN in the clinical practice settings and none of them were undertaken in Middle-East countries and in Iranian patients with T1D receiving routine care [10–19]. Earlier studies on the association between patient's characteristics and a higher incidence of HTN have evaluated nondiabetic populations or people with T2D, but rarely patients with T1D [20–26]. Accurate information regarding the incidence of HTN and associated risk factors in people with T1D is important in the prevention or delaying of its development and of the cardiovascular damage caused by this complication. Information on risk factors of incident HTN can lead to the identification of patients with T1D who may have more difficulty controlling their HTN.

The objective of this report, therefore, was to estimate the incidence of and risk factors of HTN in patients with T1D in routine clinical care. This study could also serve as a platform for future comparison with other studies and with the results obtained in other parts of Iran.

## 2. Patients and Methods

### 2.1. Study Population and Data Collection

This was a prospective registry analysis that used data from the clinical information system at Isfahan Endocrine and Metabolism Research Center, Iran, a continuing data collection program in central Iran to collect, analyze, and disseminate data in a standardized manner. Clinical data were collected for all consecutive patients at the first attendance and at review consultations using standard encounter forms. These included an assessment of the ocular fundus, lens, limbs, and blood pressure (BP) and creation of a problem list by the clinician. The following variables were examined at the time of each examination: age, age at diagnosis, duration of diabetes, height, weight, fasting plasma glucose (FPG), glycosylated hemoglobin (HbA1c), urine protein, triglyceride, cholesterol, low-density lipoprotein cholesterol (LDLC), high-density lipoprotein cholesterol (HDLC) and serum creatinine, and reporting of smoking as part of a completed questionnaire on demography, family history and smoking by the patient, and BP.

All patients were referred for the diabetes education program after the start of the therapy by trained nutritionists. The diabetes education classes included six 2 h classes emphasizing the importance of carbohydrate counting, exercise, oral and injectable medications, and microvascular and macrovascular complications of diabetes. The mechanisms of actions of diabetes medications along with proper dosing and use were reviewed, the definition and proper treatment for hypoglycemia were described, and the importance of exercise and proper foot care was explained. A computerized patient registry provided data on patient characteristics, medications, and laboratory values.

The detailed data collection methods of the Isfahan Endocrine and Metabolism Research Center outpatient clinics have been described previously [27].

## 3. Ethics Statement

The study protocol followed the Iranian government's ethical guidelines for epidemiological studies in accordance with the current version of the Declaration of Helsinki. Isfahan Endocrine and Metabolism Research Center ethical committee approval was granted. This study was based on a routine medical procedure, and additional written consent was not required. The data was processed and analyzed by authorized medical personnel only, the patients remained anonymous, and the information was deidentified prior to analysis.

## 4. Measurements

Height and weight were measured with subjects in light clothes and without shoes using standard apparatus. Weight was measured to the nearest 0.1 kg on a calibrated beam scale. Height was measured to the nearest 0.5 cm with a measuring tape. Resting systolic (phase I) and diastolic (Phase V) BP were recorded at each examination by a physician with the participants in a sitting position with their legs not crossed and the feet placed firmly on the floor, upon resting in this position for at least 10 min using a mercury column sphygmomanometer and appropriately sized cuffs. Average BP was calculated from the two consecutive measurements. FPG was measured using the glucose oxidase method. The estimated glomerular filtration rate (eGFR) was calculated using the Modification of Diet in Renal Disease (MDRD) formula [28]: eGFR = 186.3 × serum creatinine in mg/dl^−1.154^  × age^−0.203^  × 0.742 (if women). The physician defined the type of diabetes using the problem list. All blood sampling procedures were performed in the central laboratory of the Isfahan Endocrine, and Metabolism Research Center.

Predictors of HTN were assessed using the following data from the patient's registration consultation: gender, age at diagnosis, age, educational level, time since diagnosis of diabetes, BMI (weight/height^2^ [kg/m^2^]), smoking status (never, current), FPG, serum creatinine, triglyceride, cholesterol, HDLC (measured using standardized procedures), LDLC (calculated by the Friedewald equation [29]), and BP (systolic and diastolic) at initial registration and number of follow-up visits and follow-up duration.

## 5. Follow-Up and Diagnosis of HTN

**Figure 1:**
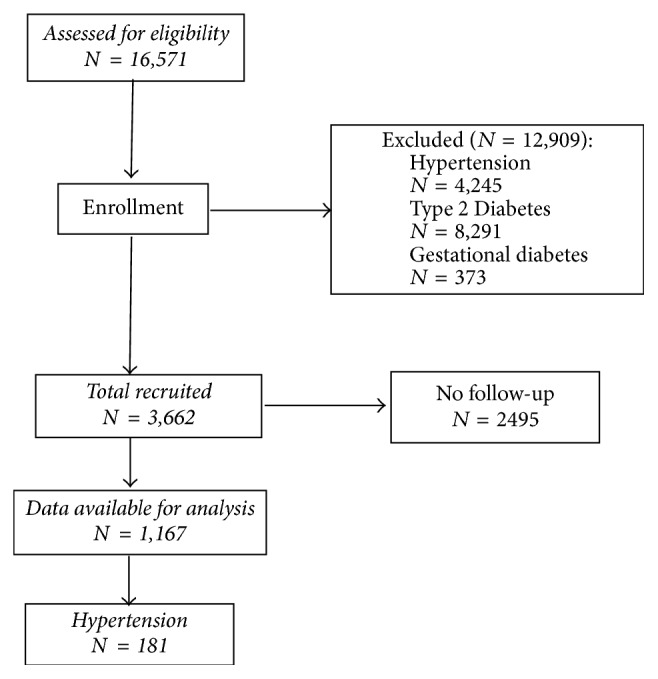
Schematic diagram of the study population.

Between 1992 and 2016, a total of 16,571 patients with gestational diabetes, T1D and T2D, were registered in the system. Women with diabetes diagnosed only during pregnancy and T2D were excluded. In order to be included in the analyses, a patient had to have at least one subsequent review during a mean (standard deviation (SD)) follow-up period of 16.2 (6.4) (range 1–25) years. However, this study uses data only for 1,167 (557 (47.7%) men and 610 (52.3%) women) HTN-free patients with T1D at baseline for whom complete data were available (Figure 1). The participants had a mean age of 20.6 (10.5) (range 1–73) years. The guidelines published by the JNC8 [30] were used as definitions for HTN and defined as a systolic BP ≥ 140 mm Hg, and/or a diastolic BP ≥ 90 mm Hg, and/or the current use of antihypertensive medication. Participants were asked as part of the medical interview whether they had ever been told by a physician that they have high or elevated BP and whether they are taking antihypertensive medication.

## 6. Analysis

Participants were followed until the occurrence of HTN, the date of the last completed follow-up, death, or end of follow-up on October 1, 2016, whichever event occurred first.

Statistical methods included the two-sided Student's *t*-test or Mann–Whitney *U* test, one-way analysis of variance (ANOVA) with Scheffe's method as the post hoc analysis or the Kruskal-Wallis test with the Dunn procedure for continuous variables, the chi-squared test for categorical variables, and multiple logistic regression. Because we had no HTN events registered in between examination cycles, crude and multivariate binary logistic regression were performed to calculate the odds ratios (ORs) with 95% confidence intervals (CI) and *P* values for new-onset HTN as the dependent variable (yes/no) using the SPSS version 18 for Windows (SPSS Inc., Chicago, IL, USA). When a new case of HTN was identified we used the examination visit date as a new case of HTN. A general linear model was used to examine the significance of trends in potential predictors of HTN and compared age- and gender-adjusted means. *P* value < 0.05 was required to confer statistical significance.

## 7. Results

### 7.1. Baseline Characteristics

The mean (SD) age of the patients included in this study was 20.6 (10.5) years, mean (SD) diabetes duration was 3.6 (4.8) years, mean (SD) BMI was 20.6 (4.8) kg/m^2^, mean (SD) HbA1c was 8.9% (2.6), mean (SD) systolic and diastolic BP were 106.3 (11.5) and 67.6 (9.8) mm Hg at baseline, and 52.3% were women. The women had significantly higher BMI than men (21.0 versus 20.3, *P* < 0.05). Age, duration of diabetes, HbA1c, age at diagnosis of diabetes, FPG, cholesterol, triglyceride, LDLC, and HDLC did not differ between the genders.

### 7.2. Prevalence and Incidence of HTN

Of the 1,293 T1D patients, 126 (70 men and 56 women) had HTN at baseline. The overall prevalence of HTN was 9.7% (95% CI: 8.2, 11.5). Prevalence of HTN was higher in men (11.2%; 95% CI: 8.7, 13.6) than women (8.4%; 95% CI: 6.4, 10.8).

During a mean of 16-year follow-up, of the 1,167 patients without HTN at baseline, 181 (15.5%) (101 men and 80 women) developed HTN in a total of 18,870 (8,922 men and 9,948 women) person-years of follow-up. The other 986 patients with T1D had not developed HTN by the end of this study period. The overall incidence of subsequent HTN was 9.6 per 1000 person-years (95% CI: 8.2, 11.0). There was a statistically increasing incidence of HTN with increasing age (*P* < 0.001): 4.9 per 1000 person-year for those aged ≤ 10 years, 8.5 for those aged 11 through 20 years, 11.5 for those aged 21 through 30 years, and 13.3 for those aged >30 years. Incidence rates were higher in men (11.3 (95% CI: 9.1, 13.5) per 1000 person-years) than women (8.0 (95% CI: 6.3, 9.8)).

### 7.3. Risk Factors

Table 1 shows the group means (SD) and proportions for those participants who did and did not develop HTN. Those who developed HTN had higher systolic (108.0 versus 106.7 mm Hg; *P* < 0.05) and diastolic (69.5 versus 67.3 mm Hg; *P* < 0.01) BP, follow-up duration (18.7 versus 15.8 yr.; *P* < 0.001), and number of follow-up visits (30.7 versus 16.9 times; *P* < 0.001) and were diagnosed with diabetes at older ages (19.3 versus 16.5 yr.; *P* < 0.001) and were older at registration (23.7 versus 20.0; *P* < 0.001). They had higher triglyceride (169.4 versus 128.1 mg/dl; *P* < 0.01) and HbA1c (9.3% versus 8.8%; *P* < 0.05) at the baseline examination. A lower proportion of those who developed HTN were the normal weight (77.3% versus 84.6%; *P* < 0.05), but higher proportion were overweight (18.2% versus 10.8%; *P* < 0.05) and were men (55.8 versus 46.2; *P* < 0.05).

To determine the influence of potential factors on HTN, univariate analysis was first performed (Table 2). Crude OR showed that those who had HTN were more likely to be older at registration and diagnosis of diabetes and had a higher duration of diabetes, HbA1c, BMI, cholesterol, and triglyceride. Age- and gender-adjusted multiple logistic regression coefficients among those free of HTN at registration showed that significant risk factors for developing HTN were male gender, older age, higher HbA1c, and triglyceride.

To determine the independent predictors of the incidence of HTN a forward (likelihood ratio) multiple logistic regression was performed. Sequential adjustment showed that only male (OR 1.48 (95% CI: 1.07, 2.05)), older age at registration (OR per SD increment, 1.03 (95% CI: 1.02, 1.04)), higher triglyceride (OR per SD increment, 1.002 (95% CI: 1.001, 1.003)), and higher systolic BP (OR per SD increment, 1.02 (95% CI: 1.003, 1.03)), significantly increased the risk of developing HTN. No other variables were significant when other covariates were considered (Table 3).

## 8. Discussion

This follow-up study of 1,167 HTN-free T1D clinic attendees found an overall incidence of HTN of 9.6 per 1000 person-years (181 patients) over an average follow-up of 16.3 years. HTN was associated with male gender, older age, higher triglycerides, and higher systolic BP. To the best of our knowledge, no other incidence rates for HTN among Iranian T1D population have been reported. Previously, many studies have examined the prevalence and incidence of HTN in the nondiabetic population [20, 21] or T2D patients [22–26] whereas information for patients with T1D are limited and reported a prevalence of 20 to 43% [10–18]. Estimates of the prevalence of HTN will depend upon the definition of the HTN used and the composition of the community examined by age, diabetes duration, and social class, making comparisons between studies of limited values. The Coronary Artery Calcification in Type 1 Diabetes Study [10] with a mean age of 37 years and duration of diabetes of 23.2 years demonstrated a higher prevalence rate of HTN among patients with T1D (43%) compared to individuals without diabetes (15%). The EURODIAB study (a prospective cohort study of people with T1D from 16 European countries) demonstrates an HTN prevalence of 24% among 3,250 patients with T1D [11] with a mean age of 32.7 years and duration of diabetes of 14.7. The prevalence of HTN in Brazil in patients with T1D with a mean age of 21.2 years and duration of diabetes of 9.6 years was 19.2% [12]. The prevalence of HTN in Pittsburgh in patients with T1D with a mean age of 29.1 years and duration of diabetes of 20.4 years at baseline was 29.0% [31]. Several possible explanations exist for our lower prevalence of HTN in patients with T1D (9.7%) compared with earlier reports. These include age and duration of diabetes (the Pittsburgh cohort had a mean age of 28 years with 20 years of duration, whereas the EURODIAB cohort had a mean age of 33 years with mean duration of 15 years at baseline; the Brazil study had a mean age of 21 years and mean duration of diabetes of 10 years compared with our mean age of 20 years and 3.6 years of duration). In addition, differences of ethnicity, clinical practice, and medical care access could also contribute to differences.

The 10-year incidence of HTN in Wisconsin Epidemiological Study of Diabetic Retinopathy in T1D was 25.9% [19]. In black persons with T1D, the 6-year incidence of HTN was 29.3% [13]. Our clinic-based incidence is lower than the values reported in Wisconsin Epidemiological Study of Diabetic Retinopathy. Lower rates in our study could have been due to a different definition of HTN, age, and duration of diabetes, and differences in medical care access and therapy might be one reason.

Klein et al. [19] reported a positive relationship between HbA1c and the incidence of HTN in T1D and suggested that glycemic control may be an effective approach for preventing the development of HTN in T1D. De Boer et al. [32] in Diabetes Control and Complications Trial (DCCT)/Epidemiology of Diabetes Interventions and Complications (EDIC) Study Research Group also reported a positive relationship between hyperglycemia and the incidence of HTN in T1D, and intensive insulin therapy reduces the long-term risk of developing HTN. In univariate analysis, the level of HbA1c ≥ 9.5%, as measured by one HbA1c determination at baseline, was associated with the development of HTN. After age- and gender-adjustment, the level of HbA1c ≥ 9.5% was found to be a predictor of incidence of HTN. After adjustment for other covariates in the multivariate analysis, the level of HbA1c was nonsignificant. Although these data have failed to confirm a relationship between metabolic control and the incidence of HTN, they in no way diminish the need for optimal glycemic control for the prevention of T1D complications, which has been well demonstrated both epidemiologically and interventionally.

Previous studies differ in relation to the importance of obesity as a risk factor. BMI but not HbA1c was significantly associated with the systolic BP in children with T1D participating in the Oxford Regional Prospective Study [33]. By multivariate analysis, however, we found no association between BMI and HTN.

While our real-life data has strengths, including its large cohort size and long-term follow-up, frequent measurement of relevant covariates, a well-characterized cohort of people with T1D, and the use of standardized protocols to measure risk factors and HTN, it also has some limitations. Because of the single center and nonrandom selection of patients, we cannot exclude the possibility of selection bias in the registry and the results may not apply to area/country groups. The study was a clinic, rather than population, based and so may not contain a clinical spectrum representative of patients with T1D in the community. Clinic-based estimates of the incidence or prevalence of complications are most likely to be affected by referral patterns. Selection bias is less likely to affect incidence rates and associations between risk factors and complications as investigated in this study. Our diagnosis of HTN is not based on a single examination but continuing examination during follow-up, using a problem list as the basis for further clinical decisions. Nevertheless, several observers made observations over the years, and problems of observer error need to be considered. It seems reasonable to assume that observer error is independent of such variables as age, gender, and duration of diabetes. If this is so, misclassification resulting from observer error will tend to reduce rather than increase the significance of differences between groups of patients. If therefore a significant difference is found between two otherwise comparable groups of patients, it is reasonable to infer that it is not due to observer error but must reflect a true difference. We could not rule out the possibility of residual confounding because of unmeasured or inaccurately measured covariates. We used a clinical definition of T1D that was assigned by clinicians and was applicable to all patients. However, autoantibody and C-peptide levels were not measured. Therefore, some patients with other types of diabetes may have been included. Nevertheless, it is important to emphasize that 86% of our patients were diagnosed before the age of 30, which supports the high probability that these patients had T1D. This is the first report of incident HTN in people with T1D in routine care in a Middle-East country and provides new data from Iran which has been underrepresented in past studies.

The results of this study highlight the need for increased attention to diagnosing HTN and vigorous blood pressure control in patients with T1D.

## Figures and Tables

**Table 1:** Age and age-adjusted means (SD) and proportions of selected baseline characteristics in 181 type 1 diabetes patients who did and 986 who did not develop hypertension at baseline.

Variables	Developed hypertension	Not developed hypertension	*P* value
	Mean (SD)	Mean (SD)	

Age (yr.)	23.7 (12.3)	20.0 (10.1)	<0.001
Age at diagnosis (yr.)	19.3 (10.6)	16.5 (9.0)	<0.001
Years since diabetes diagnosis	3.6 (0.33)	3.6 (0.14)	0.981
Number of follow-up visits	28.9 (24.8)	17.3 (18.6)	<0.001
Height (cm)	155.1 (15.5)	155.2 (18.6)	0.989
Weight (kg)	50.8 (16.3)	51.2 (18.0)	0.781
Body mass index (kg/m^2^)	20.4 (4.6)	20.6 (4.9)	0.651
Follow-up duration (yr.)	18.7 (5.5)	15.8 (6.4)	<0.001
Estimated glomerular filtration rate (eGFR) (mL/min)	120.3 (38.7)	117.7 (44.6)	0.442
Glomerular filtration rate (GFR) (mL/min)	82.6 (30.1)	82.1 (42.8)	0.884
Systolic BP (mmHg)	108.0 (11.8)	106.8 (11.3)	0.018
Diastolic BP (mmHg)	69.5 (9.7)	67.3 (9.8)	0.006
Baseline fasting glucose (mg/dl)	198.3 (98.7)	198.7 (100.5)	0.952
Creatinine (*μ*M/l)	0.83 (0.24)	0.86 (0.47)	0.428
HbA1c (%)	9.3 (2.5)	8.8 (2.6)	0.021
Triglyceride (mg/dl)	169.4 (198.8)	128.1 (105.2)	<0.001
Cholesterol (mg/dl)	186.9 (46.9)	182.8 (43.6)	0.254
HDL cholesterol (mg/dl)	47.5 (11.4)	47.1 (11.9)	0.819
LDL cholesterol (mg/dl)	111.7 (37.1)	107.7 (38.8)	0.472

	Number (%)	Number (%)	

Men	101 (55.8)	456 (46.2)	0.019
Therapeutic regimen %			
Noninsulin	31 (17.1)	128 (12.9)	0.449
Insulin	150 (82.9)	862 (87.0)	-
Education %			
Less than high school	106 (62.0)	551 (60.3)	0.636
High school	42 (24.6)	253 (27.5)	-
College graduate	23 (13.5)	112 (12.2)	-
Glycated hemoglobin %			
<7%	27 (19.7)	177 (26.7)	0.117
7%–9.5%	47 (34.3)	236 (35.6)	-
>9.5%	63 (46.0)	249 (37.6)	-
Weight category %			
Normal weight (BMI < 25.0 kg/m^2^)	136 (77.3)	805 (84.6)	0.022
Overweight (BMI 25–29.9 kg/m^2^)	32 (18.2)	103 (10.8)	-
Obese (BMI ≥ 30.0 kg/m^2^)	8 (4.5)	44 (4.6)	-

*Note*. Total of each variable may vary because of missing value. Differences in the mean or percentage values of variables between participants who developed and did not develop hypertension.

**Table 2:** Incidence rates of hypertension by baseline variables.

Variables	At risk (number)	Cases (number)	Person-year	Incidence per 1000 person-year	Crude Odds ratio (95% CI)	Age- and gender-adjusted odds ratio (95% CI)^†^
Gender						
Female	610	80	9948	8.0	1.00	1.00
Male	557	101	8922	11.3	1.47(1.07,2.02)^*∗*^	1.48(1.07,2.05)^*∗*^
Age at registration (yr.)						
≤10	160	14	2865	4.9	1.00	-
11–20	496	69	8150	8.5	1.69 (0.92,3.08)	-
21–30	342	57	4951	11.5	2.09 (1.13,3.87)^*∗*^	-
>30	163	39	2928	13.3	3.28 (1.70, 6.73)^*∗∗∗*^	-
Age at diagnosis (yr.)						
≤10	280	35	4969	7.0	1.00	1.00
11–20	534	72	8468	8.5	1.09 (0.71,1.68)	0.84 (0.53,1.36)
21–30	250	48	3722	12.9	1.66(1.04,2.67)^*∗*^	0.95 (0.50,1.80)
>30	84	21	1527	13.8	2.33(1.27,4.29)^*∗∗*^	0.84 (0.30,2.36)
Duration of diabetes (yr.)						
≤1	659	90	10198	8.8	1.00	1.00
2-3	179	29	2991	9.7	1.22 (0.78,1.93)	1.13 (0.71,1.80)
4-5	90	14	1654	8.5	1.16 (0.63,2.15)	0.94 (0.50,1.79)
6–10	119	22	2114	10.4	1.43 (0.86,2.40)	1.23 (0.72,2.10)
>10	110	23	1787	12.9	1.87(1.09,3.19)^*∗*^	1.13 (0.64,2.01)
Fasting blood glucose (mg/dl)						
<100	201	33	3294	10.0	1.00	1.00
100–126	141	22	2185	10.1	0.94 (0.52,1.70)	0.92 (0.51,1.66)
≥126	813	126	13267	9.5	0.93 (0.61,1.42)	0.89 (0.58,1.36)
HbA1 (%)						
<7.0	204	27	2430	11.1	1.00	1.00
7.0–9.5	283	47	4065	11.6	1.30 (0.78,2.19)	1.30 (0.77,2.18)
≥9.5	312	63	4665	13.5	1.66(1.02,2.71)^*∗*^	1.72(1.04,2.82)^*∗*^
BMI (Kg/m^2^)						
<25	941	136	15509	8.8	1.00	1.00
25–30	135	32	2077	14.4	1.84(1.19,2.85)^*∗∗*^	1.54 (0.96,2.45)
>30	52	8	650	12.3	1.08 (0.50,2.34)	0.92 (0.40,2.17)
Creatinine (*μ*M/l)						
≤1.2	914	156	14291	10.9	1.00	1.00
>1.2	47	11	800	13.8	1.49 (0.74,2.98)	1.12 (0.54,2.30)
Cholesterol (mg/dl)						
<200	732	106	11297	9.4	1.00	1.00
≥200	355	73	6442	11.3	1.53(1.10,2.13)^*∗*^	1.37 (0.98,1.93)
Triglyceride (mg/dl)						
<150	806	118	13048	9.0	1.00	1.00
≥150	275	61	4581	13.3	1.66(1.18,2.35)^*∗∗*^	1.45(1.01,2.08)^*∗*^

Total number of person-years and at risk is not the same for each variable because of missing values. ^*∗*^*P* < 0.5, ^*∗∗*^*P* < 0.01, ^*∗∗∗*^*P*<0.001. ^†^Odds ratios (with 95% CI) calculated by multiple logistic regression.

**Table 3:** Impact of sequential adjusted risk factors related to mean 16-year incidence of hypertension for patients with type 1 diabetes.

Variables	Odds ratio (95% confidence interval)	*P* value
Age (yr.)	1.03 (1.02, 1.04)	<0.001
Adjusted for age and gender		
Female	1.00	
Male	1.48 (1.07, 2.05)	0.017
Adjusted for all above and systolic BP (mm Hg)	1.02 (1.003, 1.03)	0.021
Adjusted for all above and triglyceride (mg/dl)	1.002 (1.001, 1.003)	0.001

## References

[1] Christlieb A. R., Warram J. H., Królewski A. S. (1981). Hypertension: the major risk factor in juvenile-onset insulin-dependent diabetics.. *Diabetes*.

[2] Parving H.-H., Andersen A., Smidt U., Svendsen P. (1983). Early aggressive antihypertensive treatment reduces rate of decline in kidney function in diabetic nephropathy. *The Lancet*.

[3] Mogensen C. E. (1982). Long-term antihypertensive treatment inhibiting progression of diabetic nephropathy. *BMJ*.

[4] Teuscher A., Schnell H., Wilson P. W. F. (1988). Incidence of diabetic retinopathy and relationship to baseline plasma glucose and blood pressure. *Diabetes Care*.

[5] Klein R., Klein B. E. K., Moss S. E., Davis M. D., DeMets D. L. (1989). Is blood pressure a predictor of the incidence or progression of diabetic retinopathy?. *JAMA Internal Medicine*.

[6] Arauz-Pacheco C., Parrott M. A., Raskin P. (2002). The treatment of hypertension in adult patients with diabetes.. *Diabetes Care*.

[7] Chobanian A. V., Bakris G. L., Black H. R. (2003). The seventh report of the joint national committee on prevention, detection, evaluation, and treatment of high blood pressure: the JNC 7 report. *The Journal of the American Medical Association*.

[8] Rossing P., Hougaard P., Borch-Johnsen K., Parving H.-H. (1996). Predictors of mortality in insulin dependent diabetes; 10 year observational follow up study. *British Medical Journal*.

[9] Forrest K. Y.-Z., Becker D. J., Kuller L. H., Wolfson S. K., Orchard T. J. (2000). Are predictors of coronary heart disease and lower-extremity arterial disease in type 1 diabetes the same?: A prospective study. *Atherosclerosis*.

[10] Maahs D. M., Kinney G. L., Wadwa P. (2005). Hypertension prevalence, awareness, treatment, and control in an adult type 1 diabetes population and a comparable general population. *Diabetes Care*.

[11] Collado-Mesa F., Colhoun H. M., Stevens L. K. (1999). Prevalence and management of hypertension in type 1 diabetes mellitus in Europe: the EURODIAB IDDM complications study. *Diabetic Medicine*.

[12] Gomes M. B., Tannus L. R. M., Matheus A. S. D. M. (2013). Prevalence, awareness, and treatment of hypertension in patients with type 1 diabetes: a nationwide multicenter study in Brazil. *International Journal of Hypertension*.

[13] Roy M. S., Janal M. N., Roy A. (2011). Medical and psychological risk factors for incident hypertension in type 1 diabetic African-Americans. *International Journal of Hypertension*.

[14] Dahl-Jørgensen K., Larsen J. R., Hanssen K. F. (2005). Atherosclerosis in childhood and adolescent type 1 diabetes: early disease, early treatment?. *Diabetologia*.

[15] Rodriguez B. L., Dabelea D., Liese A. D. (2010). Prevalence and correlates of elevated blood pressure in youth with diabetes mellitus: The search for diabetes in youth study. *Journal of Pediatrics*.

[16] Schwab K. O., Doerfer J., Marg W., Schober E., Holl R. W. (2010). Characterization of 33 488 children and adolescents with type 1 diabetes based on the gender-specific increase of cardiovascular risk factors. *Pediatric Diabetes*.

[17] van Vliet M., Van der Heyden J. C., Diamant M. (2010). Overweight is highly prevalent in children with type 1 diabetes and associates with cardiometabolic risk. *Journal of Pediatrics*.

[18] Margeirsdottir H. D., Larsen J. R., Brunborg C., Øverby N. C., Dahl-Jørgensen K. (2008). High prevalence of cardiovascular risk factors in children and adolescents with type 1 diabetes: A population-based study. *Diabetologia*.

[19] Klein R., Klein B. E. K., Lee K. E., Cruickshanks K. J., Moss S. E. (1996). The incidence of hypertension in insulin-dependent diabetes. *JAMA Internal Medicine*.

[20] Hajjar I., Kotchen T. A. (2003). Trends in prevalence, awareness, treatment, and control of hypertension in the United States, 1988–2000. *Journal of the American Medical Association*.

[21] Wolf-Maier K., Cooper R. S., Kramer H. (2004). Hypertension treatment and control in five european countries, Canada, and the United States. *Hypertension*.

[22] Geiss L. S., Rolka D. B., Engelgau M. M. (2002). Elevated blood pressure among U.S. adults with diabetes, 1988-1994. *American Journal of Preventive Medicine*.

[23] Barzilay J. I., Jones C. L., Davis B. R. (2001). Baseline characteristics of the diabetic participants in the antihypertensive and lipid-lowering treatment to prevent heart attack trial (ALLHAT). *Diabetes Care*.

[24] Gnasso A., Calindro M. C., Carallo C. (1997). Awareness, treatment and control of hyperlipidaemia, hypertension and diabetes mellitus in a selected population of southern Italy. *European Journal of Epidemiology*.

[25] Donnelly R., Molyneaux L., McGill M., Yue D. K. (1997). Detection and treatment of hypertension in patients with non-insulin-dependent diabetes mellitus: does the ‘rule of halves’ apply to a diabetic population?. *Diabetes Research and Clinical Practice*.

[26] Berlowitz D. R., Ash A. S., Hickey E. C., Glickman M., Friedman R., Kader B. (2003). Hypertension management in patients with diabetes: The need for more aggressive therapy. *Diabetes Care*.

[27] Janghorbani M., Amini M., Ghanbari H., Safaiee H. (2003). Incidence of and risk factors for diabetic retinopathy in Isfahan, Iran. *Ophthalmic Epidemiology*.

[28] Levey A. S., Coresh J., Greene T. (2006). Using standardized serum creatinine values in the modification of diet in renal disease study equation for estimating glomerular filtration rate. *Annals of Internal Medicine*.

[29] Friedewald W. T., Levy R. I., Fredrickson D. S. (1972). Estimation of the concentration of low-density lipoprotein cholesterol in plasma, without use of the preparative ultracentrifuge. *Clinical Chemistry*.

[30] James P. A., Oparil S., Carter B. L. (2014). 2014 Evidence-based guideline for the management of high blood pressure in adults: report from the panel members appointed to the Eighth Joint National Committee (JNC 8). *Journal of the American Medical Association*.

[31] Zgibor J. C., Wilson R. R., Orchard T. J. (2005). Has control of hypercholesterolemia and hypertension in type 1 diabetes improved over time?. *Diabetes Care*.

[32] De Boer I. H., Kestenbaum B., Rue T. C. (2008). Insulin therapy, hyperglycemia, and hypertension in type 1 diabetes mellitus. *JAMA Internal Medicine*.

[33] Schultz C. J., Neil H. A., Dalton R. N., Konopelska Bahu T., Dunger D. B. (2001). Blood pressure does not rise before the onset of microalbuminuria in children followed from diagnosis of type 1 diabetes. Oxford Regional Prospective Study Group. *Diabetes Care*.

